# Nitrogen Source Matters: High NH_4_/NO_3_ Ratio Reduces Cannabinoids, Terpenoids, and Yield in Medical Cannabis

**DOI:** 10.3389/fpls.2022.830224

**Published:** 2022-06-01

**Authors:** Avia Saloner, Nirit Bernstein

**Affiliations:** ^1^Institute of Soil, Water and Environmental Sciences, Volcani Center, Rishon LeTsiyon, Israel; ^2^The Robert H. Smith Faculty of Agriculture, The Hebrew University of Jerusalem, Rehovot, Israel

**Keywords:** cannabis, fertilizer, nitrogen, ammonium, nitrate, NO_3_, NH_4_, growth

## Abstract

The N form supplied to the plant, ammonium (NH_4_^+^) or nitrate (NO_3_^–^), is a major factor determining the impact of N nutrition on plant function and metabolic responses. We have hypothesized that the ratio of NH_4_/NO_3_ supplied to cannabis plants affects the physiological function and the biosynthesis of cannabinoids and terpenoids, which are major factors in the cannabis industry. To evaluate the hypothesis we examined the impact of five supply ratios of NH_4_/NO_3_ (0, 10, 30, 50, and 100% N-NH_4_^+^, under a uniform level of 200 mg L^–1^ N) on plant response. The plants were grown in pots, under controlled environment conditions. The results revealed high sensitivity of cannabinoid and terpenoid concentrations and plant function to NH_4_/NO_3_ ratio, thus supporting the hypothesis. The increase in NH_4_ supply generally caused an adverse response: Secondary metabolite production, inflorescence yield, plant height, inflorescence length, transpiration and photosynthesis rates, stomatal conductance, and chlorophyll content, were highest under NO_3_ nutrition when no NH_4_ was supplied. Ratios of 10–30% NH_4_ did not substantially impair secondary metabolism and plant function, but produced smaller inflorescences and lower inflorescence yield compared with only NO_3_ nutrition. Under a level of 50% NH_4_, the plants demonstrated toxicity symptoms, which appeared only at late stages of plant maturation, and 100% NH_4_ induced substantial plant damage, resulting in plant death. This study demonstrates a dramatic impact of N form on cannabis plant function and production, with a 46% decrease in inflorescence yield with the increase in NH_4_ supply from 0 to 50%. Yet, moderate levels of 10–30% NH_4_ are suitable for medical cannabis cultivation, as they do not damage plant function and show only little adverse influence on yield and cannabinoid production. Higher NH_4_/NO_3_ ratios, containing above 30% NH_4_, are not recommended since they increase the potential for a severe and fatal NH_4_ toxicity damage.

## Introduction

Cannabis (*Cannabis sativa* L.) is used by humanity as a medicinal crop since ancient times. In recent years, due to increasing awareness of the plants’ potential for modern medicine, its cultivation is spreading worldwide for the rapidly evolving medical cannabis industry (Decorte and Potter, 2015; Chouvy, 2019). Drug-type medical cannabis plants yield inflorescences rich in hundreds of phytochemicals such as cannabinoids, terpenoids, and flavonoids, which are the source of the plants’ biological activity (Russo et al., 2003; Andre et al., 2016; Shapira et al., 2019). The biosynthesis of these secondary metabolites is affected by environmental and cultivation conditions (Magagnini et al., 2018; Bernstein et al., 2019a,^2005^; Danziger and Bernstein, 2021a,b; Rodriguez-Morrison et al., 2021; Westmoreland et al., 2021); and the increasing demand by the pharmacological industry for a high-quality chemically standardized plant product requires understanding of the plant physiological and metabolic responses to exogenous factors, which is very limited today.

We have recently identified a sensitivity of cannabinoid and terpenoid production in drug-type (medical) cannabis to mineral nutrition, including N (Saloner and Bernstein, 2021) and P status (Shiponi and Bernstein, 2021b), and humic acid supplementation (Bernstein et al., 2019b). Progress was also made in understanding the nutritional requirements of the cannabis plant at the vegetative phase of development (Saloner et al., 2019; Saloner and Bernstein, 2020; Shiponi and Bernstein, 2021a), plant response to organic fertilization and interaction between nutrients (Caplan et al., 2017; Bevan et al., 2021), plant architectural manipulation (Danziger and Bernstein, 2021b,c), and drought stress (Caplan et al., 2019). The current study was undertaken to study the effects of N assimilation from an ammonium (NH_4_^+^) vs. nitrate (NO_3_^–^) N source on medical cannabis morpho-development, physiology, and secondary metabolism.

Nitrogen uptake by plants is mostly restricted to the uptake of NO_3_^–^ or NH_4_^+^ (Gerendás et al., 1997; Hawkesford et al., 2012). Following uptake, the N ions assimilates into amino-acids to build proteins and other N metabolites (Lea and Morot-Gaudry, 2001). As NO_3_ needs to be initially reduced to NH_4_ in order to be assimilated, and NO_3_ uptake and reduction are energy-consuming processes (Crawford, 1995; Ohyama, 2010), NO_3_ metabolism is less efficient energetically than NH_4_ metabolism (Jones and Jacobsen, 2005; Britto and Kronzucker, 2013). As NO_3_^–^ is an anion, and NH_4_^+^ is a cation, the electrochemical mechanisms for their uptake into roots are different and induce different impacts on the plant and its environment. NO_3_ may inhibit root uptake and accumulation of other nutrients (Anjana and Iqbal, 2007; White, 2012), and similarly, NH_4_ may compete with other cations for root uptake and was demonstrated to inhibit K, Ca, and Mg uptake (Cox and Reisenauer, 1977; Rayar and van Hai, 1977; Bernstein et al., 2005, 2011; White, 2012). Furthermore, the supplied N form may have a direct impact on the uptake, translocation, and accumulation of N in the plant (Britto and Kronzucker, 2002, 2005; Zhang et al., 2019). Another issue associated with N uptake is the different impact of the two N forms on the pH of the rhizosphere. When the root absorbs NH_4_, H^+^ is released to the rhizosphere, causing rhizosphere acidification, accompanied by organic acid biosynthesis in the root cells (Britto and Kronzucker, 2005; Neumann and Römheld, 2012). Conversely, NO_3_ influx causes rhizosphere alkalinization, accompanied by organic acid biodegradation (Crawford and Glass, 1998; Neumann and Römheld, 2012). Hence, NH_4_/NO_3_ ratio has a critical influence on the plant energy status, plant and rhizosphere pH adjustment, mineral uptake, and other key metabolic and regulatory processes that determine the plants’ physiological and horticultural performances.

Although N is a macronutrient essential for all plants in high amounts, N oversupply may severely impact plant metabolism and function, and in some cases may be lethal (Britto et al., 2001; Albornoz, 2016) by mechanisms that involve mainly NO_3_ and NH_4_ uptake and assimilation by the root (Britto and Kronzucker, 2002; Hawkesford et al., 2012; White, 2012). Therefore, an oversupply of NO_3_ or NH_4_ may be fatal to plants, and the nutritional demands of crops should be studied. No information is available today about the effect of NH_4_/NO_3_ ratio and NO_3_ or NH_4_ toxicity on plant function and secondary metabolism in *C. sativa*.

Plant species may differ in preference for NO_3_-based nutrition or NH_4_-based nutrition, considering their ability to utilize and absorb NO_3_, regulate the rhizosphere pH, and cope with a high supply of NH_4_ (Feng and Barker, 1990; Britto and Kronzucker, 2002). As a result of the negative and positive physiological effects of each NO_3_ and NH_4_, most agricultural plants perform best under a combined NH_4_ and NO_3_ supply, at the range of 10–30% NH_4_/NO_3_ ratio (Errebhi and Wilcox, 1990a; Ben-Oliel et al., 2005; González García et al., 2009). Moreover, studies conducted on different plant species indicate that supplying to the plants both NH_4_ and NO_3_ compared with only one of these N forms, could induce an increase in plant secondary metabolism (Fritz et al., 2006; Sharafzadeh et al., 2011; Saadatian et al., 2014; Zhu et al., 2014; Zhang et al., 2019). Since the response of “drug-type” medical cannabis in particular and *C. sativa* in general to NO_3_ and NH_4_ supply are yet unknown, it is difficult to predict the NH_4_/NO_3_ ratio required for optimal cannabis cultivation.

We have recently demonstrated that in medical “drug-type” cannabis, optimal plant function and development, are achieved under 160 mg L^–1^ N at both the vegetative growth phase (Saloner and Bernstein, 2020) and the flowering stage (Saloner and Bernstein, 2020), with a negative correlation between N accumulation and plant secondary metabolism. Cannabinoid and terpenoid production was found to be suppressed by the elevation of N supply, while the plant physiological function and inflorescence yield increase with the elevation of N supply up to 160 mg L^–1^ N (Saloner and Bernstein, 2021). Regardless of the recent considerable progress in our understanding of medical cannabis N nutritional requirements, all the available studies were conducted using a uniform ratio of NH_4_/NO_3_, and the effect of NH_4_/NO_3_ ratio on medical cannabis remains unknown.

In the present study, we therefore focused on the impact of the ratio between the N sources supplied to the plants (NH_4_/NO_3_ ratio) on the development, physiology, yield, and secondary metabolism of “drug-type” medical cannabis. The hypothesis guiding the workplan was that NH_4_/NO_3_ ratio elicits changes to the cannabinoid and terpenoid biosynthesis, as well as affects developmental and physiological characteristics. The project aimed to determine the optimal NH_4_/NO_3_ ratio for medical cannabis production, and to analyze possible effects of non-optimal ratios. To evaluate the hypothesis, we studied the impacts of five NH_4_/NO_3_ ratios (0, 10, 30, 50, and 100% N-NH_4_, and the remaining N was supplied as N-NO_3_), under a uniform N level, on medical cannabis. The results of this study may serve as guidelines for medical cannabis cultivation as part of a pressing need to understand medical cannabis nutritional requirements for a control of yield and secondary metabolites production.

## Materials and Methods

### Plant Material and Growing Conditions

Medical cannabis (*C. sativa* L.) plants of the cultivar “Annapurna 2” (Canndoc LTD., Herzliya, Israel), a certified cultivar for commercial medical use in Israel, were used as a model plant in this study. Plants were propagated from rooted cuttings in coconut fiber plugs (Jiffy International AS, Kristiansand, Norway) (Saloner and Bernstein, 2021). Twenty eight days following cutting from the mother plants, the developed plants were replanted to 3 L plastic pots, in perlite 2-1-2 cultivation media (Agrekal, Habonim, Israel). Perlite was selected as it is a relatively inert media that does not change chemical properties of the solution and is therefore often used for mineral nutrition studies. At the first week after planting (during the week of vegetative growth) before the initiation of the nutritional treatments, a uniform fertigation regime was practiced under long-photoperiod (18/6 h light/dark) in a controlled environment growing room. The plants were irrigated daily to allow 30% drainage; the fertigation followed the optimal N and K regimes recently developed by us for the vegetative growth phase (Saloner et al., 2019; Saloner and Bernstein, 2020), i.e., 160 mg L^–1^ N and 175 mg L^–1^ K; temperature was regulated to 27°C, relative humidity to 58%, and light intensity was 400 μmol m^–2^ s^–1^ supplied by Metal Halide bulbs (Solis Tek Inc., Carson, CA, United States). Uniform plants were then selected for the experiment, and randomly divided into treatment groups of increasing N-NH_4_^+^ supply: 0, 10, 30, 50, and 100% N-NH_4_^+^ (and a corresponding decrease in N-NO_3_^–^ supply; Supplementary Table 1), five replicated plants per treatment. The remaining N was supplied as N-NO_3_^–^, to a final uniform level of 200 mg L^–1^ N in all treatments. This level was previously demonstrated to be within the optimal range for medical cannabis cultivation (Bevan et al., 2021; Saloner and Bernstein, 2021). Throughout the remainder of the experiment, the plants were cultivated under a short photoperiod (12/12 h light/dark) for the induction of inflorescence development. Light at the short-day period was supplied by High-Pressure Sodium bulbs (980 μmol m^–2^ s^–1^, Greenlab by Hydrogarden, Petah Tikva, Israel). Average daily temperature in the cultivation room was 25°C, and relative humidity was 38% and 70% day/night, respectively. Irrigation was supplied *via* 1 L h^–1^ discharge-regulated drippers (Netafim, Tel-Aviv, Israel), one dripper per pot, 500–750 ml/pot/day, to allow 30% drainage. Mineral nutrients were supplied dissolved in the irrigation solution at each irrigation, from final (pre-mixed) solutions. At the last week before harvest the plants were irrigated with distilled water without fertilizers as is routinely practiced in the commercial cultivation of medical cannabis. The irrigation solution contained (in mg L^–1^): 110 K^+^, 59 P-PO_4_^2–^, 57 Ca^2+^, 38 Mg^2+^, 112 S-SO_4_^2–^, 39 Na^+^, 51 Cl^–^, 1.31 Fe^2+^, 0.6 Mn^2+^, 0.3 Zn^2+^, 0.13 Cu^2+^,0.1 B^3+^, and 0.003 Mo^2+^. The concentrations in mM are detailed in Supplementary Table 1. N was supplied in a constant concentration of 14.3 mM [e.g., 200 mg L^–1^ (ppm)]. The treatments included different ratios of N-NH_4_^+^/N-NO_3_^–^: 0, 10, 30, 50, and 100% of the total N. Zinc, Cu, and Mn were supplied chelated with EDTA, and Fe was chelated with EDDHSA. Boron and Mo were added from the fertilizers B-7000 and Bar-Koret, respectively (Israel chemicals, Tel-Aviv, Israel). The concentrations of the three major macronutrients used for fertigation in the present study (200 mg L^–1^ N, 60 mg L^–1^ P, and 100 mg L^–1^ K), were selected to be within the optimal range for medical cannabis plant development and function, following on previous studies that reported plant responses to a range of supply rates (Saloner et al., 2019; Bevan et al., 2021; Saloner and Bernstein, 2021; Shiponi and Bernstein, 2021b). The irrigation solution was made once weekly and the pH of the solution was adjusted to 5.6–6.0. Initial analyses ensured that the concentration of the micro and macronutrients, including the two N species tested in the study (ammonium and nitrate) were steady over time in the irrigation solution. The experiment was arranged in a complete randomized design; planting density was five plants/m^2^; all measurements were conducted for five replicated plants per treatment following the experimental design.

### Inorganic Mineral Analysis and Physiological Parameters

Concentrations of mineral nutrients in the plant organs were analyzed at the termination of the experiment, 59 days after the initiation of the NH_4_/NO_3_ treatments. The inorganic mineral analysis was performed following Saloner and Bernstein (2021, 2020).

Electrical conductivity (EC) and pH of the irrigation and leachate solutions were measured once a week. The obtained pH values are presented in Supplementary Figure 2; EC of the irrigation solution is presented in Supplementary Table 1; and EC of the leachate solutions was steady and higher than of the irrigation solution by 7–16% in all treatments.

The plants were sampled for physiological analyses 45 days after the initiation of the fertigation treatments. Gas exchange parameters are known to change during plant development (Yep et al., 2020; Shiponi and Bernstein, 2021a,b) this day was selected for the measurements as treatments affects that were cumulative over time became prominent at this time, while the gas exchange activity was still very active. Determination of photosynthetic pigments, osmotic potential, membrane leakage, relative water content (RWC), photosynthesis rate, transpiration rate, stomatal conductance, intercellular CO_2_ concentration, and water use efficiency (WUEi) were performed following Saloner and Bernstein (2020).

### Plant Development and Biomass

Plant architecture and development (Plant height, stem diameter, inflorescence length, and the number of nodes on the main stem) were measured one week prior to the termination of the experiment. The measurements were performed as described by Saloner and Bernstein (2021). Biomass accumulation in the plant organs (leaves, stems, inflorescences, inflorescence leaves, and roots) was measured by destructive sampling at the termination of the experiment, 59 days following the initiation of the NH_4_/NO_3_ treatments. Dry weights were determined after drying at 64°C for 48 h (128 h for the inflorescences).

### Cannabinoid and Terpenoid Analysis

Cannabinoid and terpenoid concentrations were evaluated in the apical (top) inflorescence of the main stem [primary inflorescence] and the apical inflorescence of the lowest first order (side) branch on the main stem [secondary inflorescence] at the termination of the experiment, 59 days following the initiation of the treatments and the short photoperiod. At that time, ∼30% of the glandular stalked-trichome heads were of amber color, which is the maturation stage acceptable for commercial harvesting in the studied cultivar. The inflorescences were wet-trimmed by hand, to separate the protruding parts of the inflorescence leaves from the inflorescence. The trimmed inflorescences and the trimmed inflorescence leaves were dried in the dark, on drying trays, at 19°C and 55% relative humidity, in an environment controlled chamber. After 14 days of drying, when the inflorescences had 10% moisture-content, the samples were packed individually in sealed plastic bags that were kept in a the dark at 25°C for 4 weeks, and opened daily for 15 min for curing before analyses.

The cannabinoid and terpenoid content in the inflorescences and inflorescence leaves were analyzed by HPLC and GCMS, correspondingly, following an extraction with ethanol ABS AR (for cannabinoids) and MTBE (for terpenoids) as is described by Saloner and Bernstein (2021). For the cannabinoid analysis, the inflorescences were ground using a manual spice-grinder, 50 mg of the ground plant material was inserted to a 50 ml centrifuge tube, 10 mL ethanol ABS AR (Gadot-Group, Netanya, Israel) was added, the tube was placed in a reciprocal shaker (1 h) and then centrifuged for 15 min (5000 rpm) (Megafuge 16, Thermo-Scientific, Waltham, MA, United States). Supernatants were filtered with 0.22 μm filter (PVDF, Bar-Naor Ltd., Ramat Gan, Israel) and cannabinoids in the extracts were analyzed by HPLC (Jasco 2000 Plus), with a PDA detector (Jasco, Tokyo, Japan). Chromatographic separation was conducted with a Luna Omega 3 μm Polar C18 column (Phenomenex, Torrance, CA United States) in isocratic mode with 75:25 (v/v) acetonitrile:water and 0.1% formic acid, at flowrate of 1.0 mL min^–1^. Calculation of cannabinoid concentrations were based on pure analytical standards: Cannabichromene (CBC), cannabichromenic acid (CBCA), cannabichromevarin (CBCV), cannabigerol (CBG), cannabigerolic acid (CBGA), cannabinol (CBN), cannabinolic acid (CBNA), cannabidiol (CBD), cannabidiolic acid (CBDA), cannabicyclol (CBL), cannabidivarin (CBDV), cannabidivarinic acid (CBDVA), tetrahydrocannabivarinic acid (THCVA) (Sigma-Aldrich, St. Louis, MO, United States); cannabicitran (CBT) (Cayman Chemical Company, PA, United States); and tetrahydrocannabinolic acid (THCA), Δ^9^-tetrahydrocannabinol (THC), Δ^8^-THC, tetrahydrocannabivarin (THCV) (Restek, Bellefonte, PA, United States). Concentrations of Δ^8^-THC, THCV, CBDV, CBDVA, CBG, CBNA, CBL, CBCV, and CBT were lower than the detection limits. Cannabichromene (CBC) concentration was in the range of 0.04–0.09% and 0.02–0.04% for the inflorescences and inflorescence leaves, respectively (Data not shown). The total weights of the four main cannabinoids in the plant, THC, CBD, CBC, and CBG, were calculated by multiplying the organ biomass by the concentration of the cannabinoids in the organ (Supplementary Figure 4), while taking into consideration differences in mass of the carboxylated vs. decarboxilated forms as of the following equations:

Total⁢THC=⁢THCA*0.877+THC

Total⁢CBD=⁢CBDA*0.877+CBD

Total⁢CBC=CBCA*0.877+CBC

Total⁢CBG=⁢CBGA*0.878+CBG

For the terpenoid analysis, one hundred mg of dried plant material was crushed in N_2_ (L). Volatiles were extracted by shaking for 2 h in 2 mL MTBE (methyl tert-butyl ether), containing ethyl myristate (100 ppm) as an internal standard. The upper MTBE layer was separated and dried with Na_2_SO_4_ and maintained at −20°C until analysis. 1 μL of the sample was injected into an GC-MSD (6890 N/5973 N, Agilent Technologies, Santa Clara, CA, United States) with Rxi-5sil ms column (30 m length × 0.25 mm i.d., 0.25 μm film thickness, stationary phase 95% dimethyl- 5% diphenyl polysiloxane). Helium (11.18 psi) was used as a carrier gas with splitless injection (250°C) and a detector temperature of 280°C. The initial temperature was 50°C for 1 min, followed by a ramp of 5°C min^–1^ to 260°C and 20°C min^–1^ up to 300°C (10 min). The MS data was acquired with a quadrupole mass detector with electron ionization at 70 eV in the range of 41–350 m/z. Identification of the compounds was conducted by comparing their relative retention indices and mass spectra with those of authentic samples or with those found in the literature and supplemented with W10N11 and QuadLib 2205 GC-MS libraries. A straight-chain alkanes blend (C7–C23) was injected into the column for the calculation of the retention indices. The amount of the compound in a sample was calculated by multiplying the peak area by the response factor of the internal standard, and dividing the result by the product of the response factor and the internal standard. As the plants in the 100% NH_4_ treatments were severely affected by NH_4_ toxicity and wilted at early stages of the experiment, their terpenoid content were not examined.

### Statistical Analyses

The experiment was performed in a random experimental design, with 5 treatments and 5 replicates per treatment. The data were analyzed by a one-way or two-way analysis of variance (ANOVA) followed by Tukey’s HSD test. Means were compared by Fisher’s least significant difference (LSD) test at 5% level of significance. The analysis was performed with the Jump package, version 9 (SAS 2015, Cary, NC, United States).

## Results

### Plant Visual Characteristics

**FIGURE 1:**
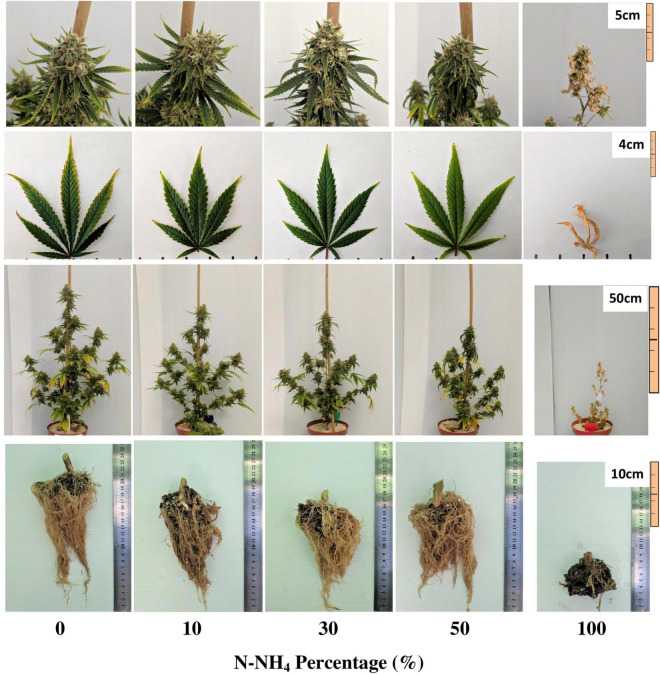
Visual appearance of the inflorescences, leaves, whole plants, and roots under increasing N-NH_4_ supply. The inflorescence images are of the apical inflorescence on the main stem, and leaf images are of the youngest, fully developed leaf located on the third branch from the plant’s top. Inflorescence, whole plant and root images were taken at maturation (harvest), leaf images were taken 10 days earlier. The brown substance at the base of the roots is a remnant of the coconut fiber plug used for rooting the cuttings at the propagation of the plants.

**FIGURE 2:**
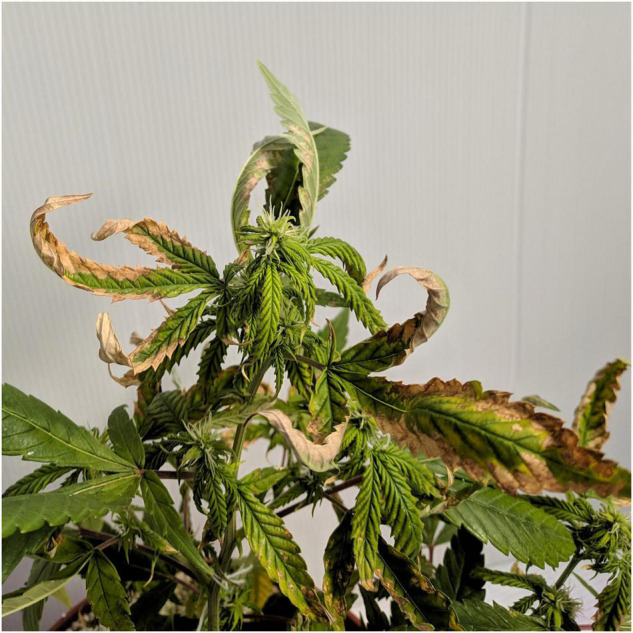
Early symptoms of NH_4_ toxicity in medical cannabis. The image was taken 17 days after the initiation of the NH_4_/NO_3_ fertigation treatments, showing the apical part of a plant receiving a 100% N-NH_4_ regime.

The visual appearance of the plant is a primary indicator of the plant physiological state. Plants in the 0–30% NH_4_ treatments appeared similar, and demonstrated adequate plant, leaf and inflorescence structure, and color (Figure 1). Nevertheless, the leaves of the 0% NH_4_ treatment developed an unfamiliar dotted chlorosis at their margins (Figure 1). The plants of the 50% NH_4_ treatment appeared normal throughout most of the experiment duration (Figure 1). However, at the last 2 weeks of the experiment, the leaves became necrotic and wilted, eventually causing death of 40% of the plants in the last week of the experiment. The plants of the 100% NH_4_ regime suffered stunted growth and toxicity symptoms already at early stages of the experiment, showing curly leaves with marginal necrosis at the plant growing tips (Figure 2). As the experiment proceeded, the 100% NH_4_ plants remained small, and the necrosis spread to all plant parts resulting in plant death (Figure 1). The visual response obtained at the areal parts of the plant is in accord with the development of the roots, which demonstrated optimal development under 0–50% NH_4_, and severe damage under 100% NH_4_ (Figure 1).

### Development and Biomass Accumulation

**FIGURE 3:**
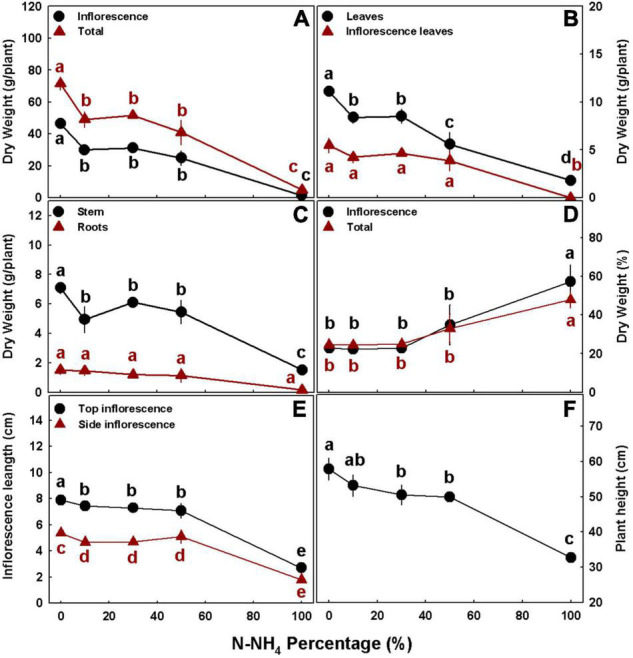
Effect of NH_4_/NO_3_ ratio on plant biomass and development in medical cannabis plants. Dry weights of inflorescences and whole plant **(A)**, leaves and inflorescence leaves **(B)**, stem and roots **(C)**, % dry weight of inflorescences and whole plant **(D)**, inflorescence length for the top (primary) inflorescence and side (secondary) inflorescence **(E)**, and plant height **(F)**. Presented data are averages ± SE (*n* = 5). Different small letters above the means represent significant differences between treatments within a curve by Tukey HSD test at α = 0.05.

In accord with the visual appearance of the plants, the numerical evaluation as well demonstrated that plant development was significantly influenced by the NH_4_/NO_3_ ratio. Plant height and inflorescence length decreased with the increase in NH_4_ percentage, demonstrating optimal growth under 0–10 and 0% NH_4_, respectively, and substantial damage under 100% NH_4_ (Figures 3E,F). The inflorescence length exhibited similar response trends to NH_4_/NO_3_ ratio for the top and side inflorescences, except that side inflorescences were significantly shorter than top inflorescences (Figure 3E). Stem diameter and the number of nodes on the main stem were also smaller in the plants receiving 100% NH_4_, and no significant differences were obtained between the other treatments (Supplementary Figure 1).

Plant biomass production generally decreased with the increase in NH_4_ supply (Figure 3). Dry biomass of all individual organs, as well as whole plant biomass, were highest under complete NO_3_ nutrition (i.e., 0% NH_4_), and lowest under 100% NH_4_ (Figures 3A–C). Inflorescence biomass, stem biomass, and whole plant biomass were highest under 0% NH_4_, significantly lower under 10–50% NH_4_, and lowest under 100% NH_4_ (Figures 3A,C). Biomass of the fan leaves demonstrated a similar trend, but under 50% NH_4_, it was significantly lower than under 10–30% NH_4_, while the NH_4_/NO_3_ regime did not significantly affect biomass of the inflorescence leaves and roots at the 0–50% NH_4_ range (Figures 3B,C). Specifically, inflorescence biomass, which may be referred to as the plant yield, decreased by 35, 32, 46, and 97% with the increase of NH_4_ supply from 0 to 10, 30, 50, and 100%, respectively (Figure 3A). Since cannabis inflorescences for marketing and consumption are dried to ∼10% moisture-content, economical yields are about 10% higher than the presented inflorescences dry biomasses. The NH_4_/NO_3_ regime also influenced the percentage of dry weight of the inflorescences and the whole plant: They were significantly higher under 100% NH_4_ nutrition compared with the lower NH_4_ treatments, amongst no significant difference was obtained (Figure 3D).

### Cannabinoid and Terpenoid Profiles

**FIGURE 4:**
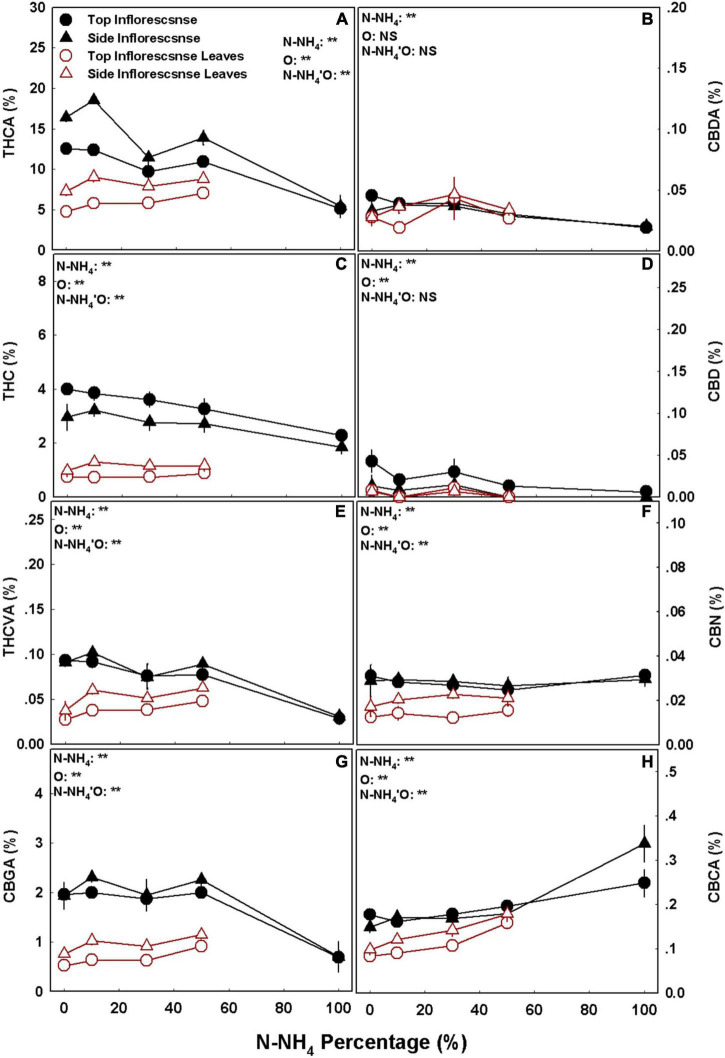
Effect of NH_4_/NO_3_ ratio on cannabinoid concentrations in medical cannabis plants. Concentrations of THCA **(A)**, CBDA **(B)**, THC **(C)**, CBD **(D)**, THCVA **(E)**, CBN **(F)**, CBGA **(G)**, and CBCA **(H)** in top (primary) and side (secondary) inflorescences and inflorescence leaves. Presented data are averages ± SE (*n* = 5). Results of two-way ANOVA indicated as **P < 0.05, *F*-test; NS, not significant *P* > 0.05, *F*-test. In the ANOVA results N-NH_4_’O represents the interaction between NH_4_/NO_3_ ratio and plant organ.

Cannabinoid concentrations in the plants were differentially affected by the NH_4_/NO_3_ ratio, as the response was cannabinoid specific and varied between organs. The accumulation of the different cannabinoids in the plant reproductive organs followed four response trends: (1) THC and CBDA concentrations in the top inflorescences, as well as THCA concentration in the side inflorescences, decreased with the increase in NH_4_ supply (Figures 4A–C). (2) The concentrations of THCVA and CBGA in both top and side inflorescence, as well as the concentration of THCA in the top inflorescence, were not significantly affected by the NH_4_/NO_3_ treatments in the range of 0–50% NH_4_, but were significantly lower under 100% NH_4_ (Figures 4A,E,G). (3) CBCA concentration in all reproductive organs, as well as THCVA and CBGA concentration in the side inflorescence leaves, were higher under high NH_4_ (100% NH_4_ for the inflorescence and 50% NH_4_ for the inflorescence leaves) compared with all other treatments (Figures 4E,G,H). (4) Except the responses mentioned above, no other significant differences in cannabinoid concentrations were obtained, as in all other examinations the cannabinoid content was not affected by the NH_4_/NO_3_ ratio (*p* > 0.05) (Figure 4). In accord with the trends mentioned above, the total amounts of major cannabinoids produced per plant decreased as well with the increase in NH_4_ supply, as the total weight of the THC forms, CBC forms, and CBG forms were higher under 0 > 10–50 > 100 percent NH_4_, while the amount of the CBD forms decreased only under 100% NH_4_ (Supplementary Figure 4).

**FIGURE 5:**
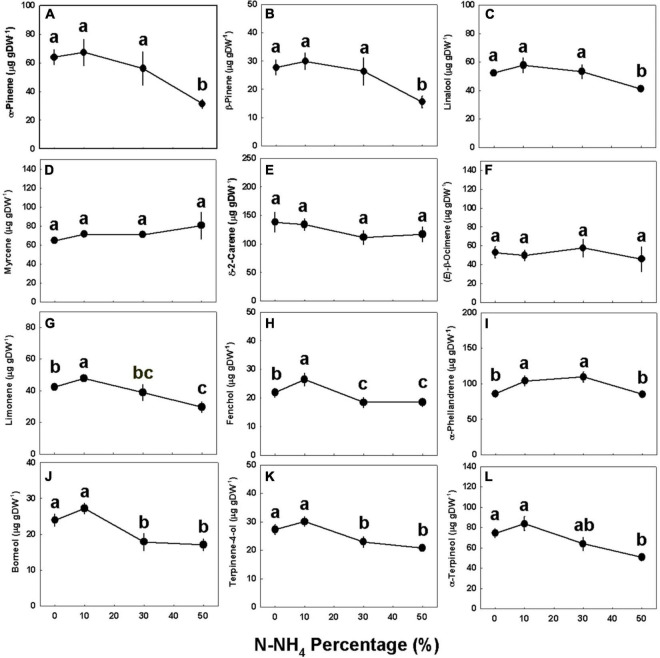
Effect of NH_4_/NO_3_ ratio on monoterpene concentration in medical cannabis plants. α-Pinene **(A)**, β-pinene **(B)**, linalool **(C)**, myrcene **(D)**, δ-2-carene **(E)**, (*E*)-β-ocimene **(F)**, limonene **(G)**, fenchol **(H)**, α-phellandrene **(I)**, borneol **(J)**, terpinen-4-ol **(K)**, and α-terpineol **(L)** concentration in the top inflorescence. Presented data are averages ± SE (*n* = 5). Different small letters above the means represent significant differences between treatments by Tukey HSD test at α = 0.05.

**FIGURE 6:**
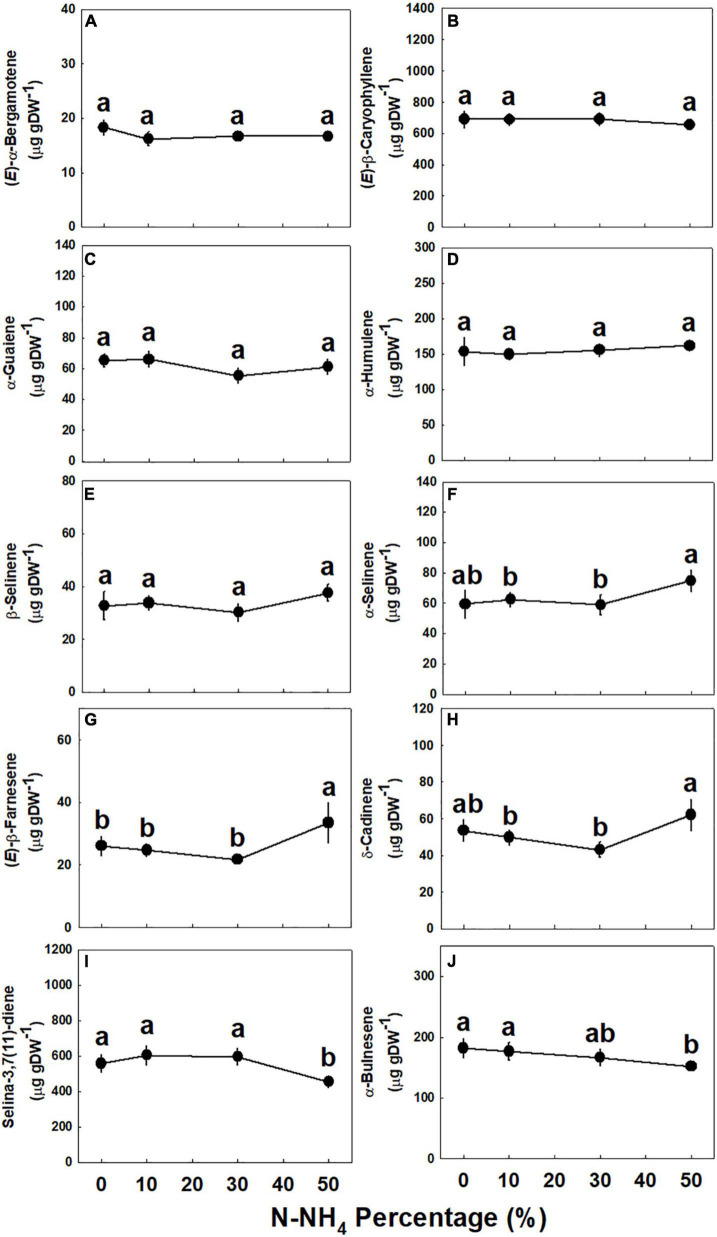
Effect of NH_4_/NO_3_ ratio on sesquiterpene concentration in the top inflorescence of medical cannabis plants. (*E*)-α-Bergamotene **(A)**, (*E*)-β-caryophyllene **(B)**, α-guaiene **(C)**, α-humulene **(D)**, β-selinene **(E)**, α-selinene **(F)**, (*E*)-β-farnesene **(G)**, δ-cadinene **(H)**, selina-3,7(11)-diene **(I)**, and α-bulnesene **(J)**. Presented data are averages ± SE (*n* = 5). Different small letters above the means represent significant differences between treatments by Tukey HSD test at α = 0.05.

Concentrations of all identified monoterpenes were highest (*p* < 0.05) under 0–10% NH_4_, except limonene, fenchol, and α-phellandrene, that had higher concentrations under 10% NH_4_ than 0% NH_4_ (Figure 5). The concentrations of myrcene, δ-2-carene, and (*E*)-β-ocimene were not affected by the NH_4_/NO_3_ ratio (Figures 5D–F); limonene, fenchol, borneol, and terpinen-4-ol were lower when NH_4_ supply was elevated above 10% (Figures 5G,H,J,K); and α-pinene, β-pinene, linalool, α-phellandrene, and α-terpineol did not decrease as NH_4_ supply was elevated to 30% and were significantly lower only under 50% NH_4_ (Figures 5A–C,I,L). The NH_4_/NO_3_ ratio had but a small effect on sesquiterpene concentrations, as the concentrations of most detected sesquiterpenes were not significantly affected by the treatments (Figure 6 and Supplementary Figure 3). Compared with 0–30% NH_4_ inputs, the concentrations of α-selinene, (*E*)-β-farnesene, and δ-cadinene were higher (*p* < 0.05) under 50% NH_4_, while the concentrations of selina-3,7(11)-diene and α-bulnesene were lower under 50% NH_4_ (Figures 6F–J). Concentrations of additional sesquiterpenes detected, revealing the same trends described above, are presented in Supplementary Figure 3.

### The Plant Ionome

**FIGURE 7:**
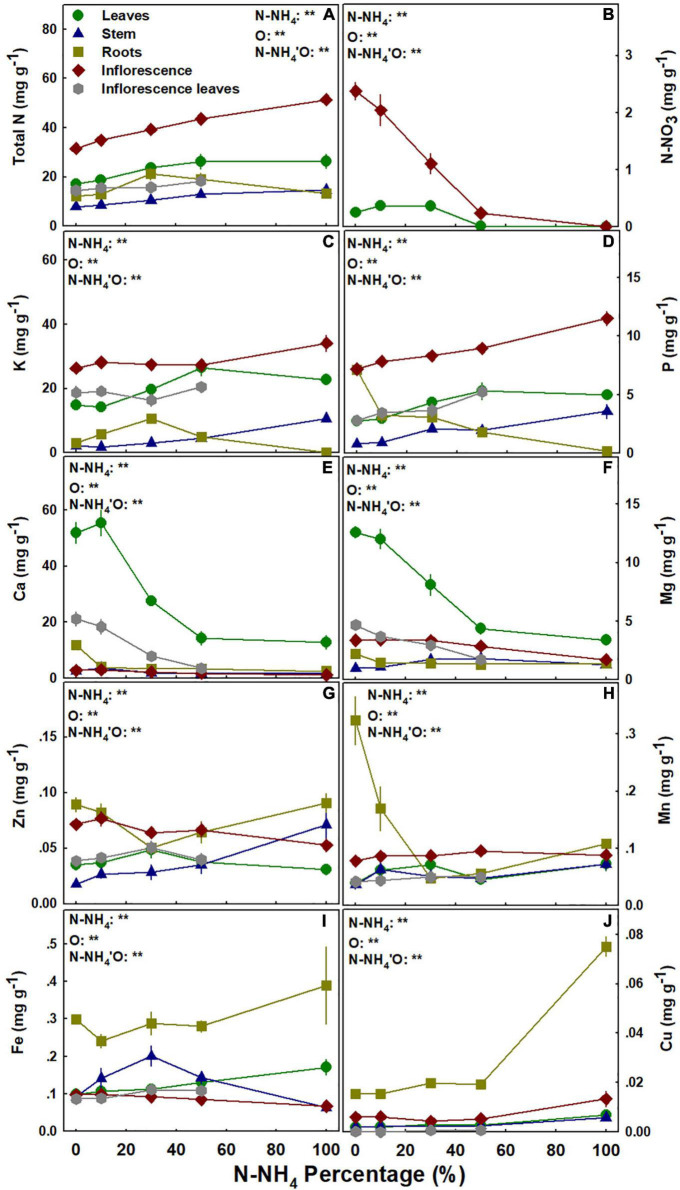
Effect of NH_4_/NO_3_ ratio on nutrient concentration in medical cannabis plants. Total N **(A)**, N-NO_3_ **(B)**, K **(C)**, P **(D)**, Ca **(E)**, Mg **(F)**, Zn **(G)**, Mn **(H)**, Fe **(I)**, and Cu **(J)**. Presented data are averages ± SE (*n* = 5). Results of two-way ANOVA indicated as **P < 0.05, *F*-test; NS, not significant *P* > 0.05, *F*-test. In the ANOVA results N-NH_4_’O represents the interaction between NH_4_/NO_3_ ratio and plant organ.

As NO_3_ is an anion and NH_4_ is a cation, it should be expected that NH_4_/NO_3_ application ratio will affect uptake of other mineral nutrients into the root and their translocation and accumulation in the plant. The impact of the NH_4_/NO_3_ ratio on the different nutrients was nutrient specific, and the response trends for plant uptake, translocation, and accumulation varied between minerals (Figure 7). Total N concentration in the stem and the inflorescences increased with the increase in NH_4_ supply (Figure 7A). N accumulation in the leaves increased as well with the elevation of NH_4_ supply, but only up to stabilization at 50% NH_4_; while N concentration in the roots was highest under 30% NH_4_ (Figure 7A). NO_3_ concentration in the leaves was higher under 0–30% NH_4_, while in the inflorescences it decreased dramatically as NO_3_ supply was restricted with the increase in NH_4_/NO_3_ ratio (Figure 7B). K concentration in the leaves was higher under 50–100% NH_4_ than under 0–10% NH_4_, while in the inflorescence it was significantly higher only under 100% NH_4_ compared to all other treatments (Figure 7C). NH_4_/NO_3_ ratio > 30% increased K concentration in the stem, and root K concentration presented a maximum accumulation curve at 30% NH_4_ (Figure 7C). P concentration in the stem, inflorescences, and inflorescence leaves increased with the increase in NH_4_ supply, while an opposite trend was obtained for the roots (Figure 7D). As was obtained for K accumulation, P concentration in the leaves was higher under 50–100% NH_4_ than under 0–10% NH_4_ (Figures 7C,D). Ca and Mg concentrations in the leaves, and Ca concentration in the inflorescences, were higher under 0–10 > 30 > 50–100 percent NH_4_ (Figures 7E,F). Ca and Mg concentrations in the stem demonstrated maximum response curves with the highest concentration at 10% and 30–50% NH_4_, respectively (Figures 7E,F). Ca concentration in the roots was higher under 0% NH_4_; Mg concentration in the roots was unaffected by the NH_4_/NO_3_ ratio; Mg concentration in the inflorescence was lower under 100% NH_4_ (Figures 7E,F); and Ca and Mg accumulation in the inflorescence leaves decreased with the increase in NH_4_ supply (Figures 7D–F). Except for P, Ca, and Mg, the accumulation of all other nutrients in the inflorescence leaves was not affected by the NH_4_/NO_3_ ratio supplied (Figure 7).

NH_4_/NO_3_ ratio had only minor effects on the concentration of micronutrients in the medical cannabis plants. In the leaves, Zn and Mn were not significantly affected by the NH_4_/NO_3_ ratio, but Fe concentration was highest under 100% NH_4_ (Figure 7). Zn concentration in the stem was highest under 100% NH_4_, while in the roots it demonstrated a minimum curve (Figure 7G). Mn concentration in the roots decreased with the elevation of NH_4_ up to 30%, but the concentration in the stem was not affected by the treatments (Figure 7H). Fe concentration in the stem demonstrated a maximum accumulation curve with the highest concentration under 30% NH_4_, while roots’ Fe was unaffected by the NH_4_/NO_3_ ratio (Figure 7I). Zn, Mn, and Fe concentrations in the inflorescence were also unaffected by the NH_4_/NO_3_ ratio (Figures 7G–I). Cu concentration in all plant organs was higher under 100% NH_4_, compared to lower NH_4_/NO_3_ ratio inputs (Figure 7J). Cu and Fe accumulated in the roots to higher concentrations than in all other plant organs (Figures 7I,J).

The pH of the irrigation solutions was similar for all treatments (Supplementary Figure 2), and as expected, pH of the leachate solution was significantly higher than the irrigation solution pH under 0% NH_4_, and lower than the irrigation solution pH under 100% NH_4_ (Supplementary Figure 2), showing acidification and alkalization of the rhizosphere under high NH_4_ and high NO_3_, respectively.

### Gas Exchange, Water Relations, and Photosynthetic Pigments

**FIGURE 8:**
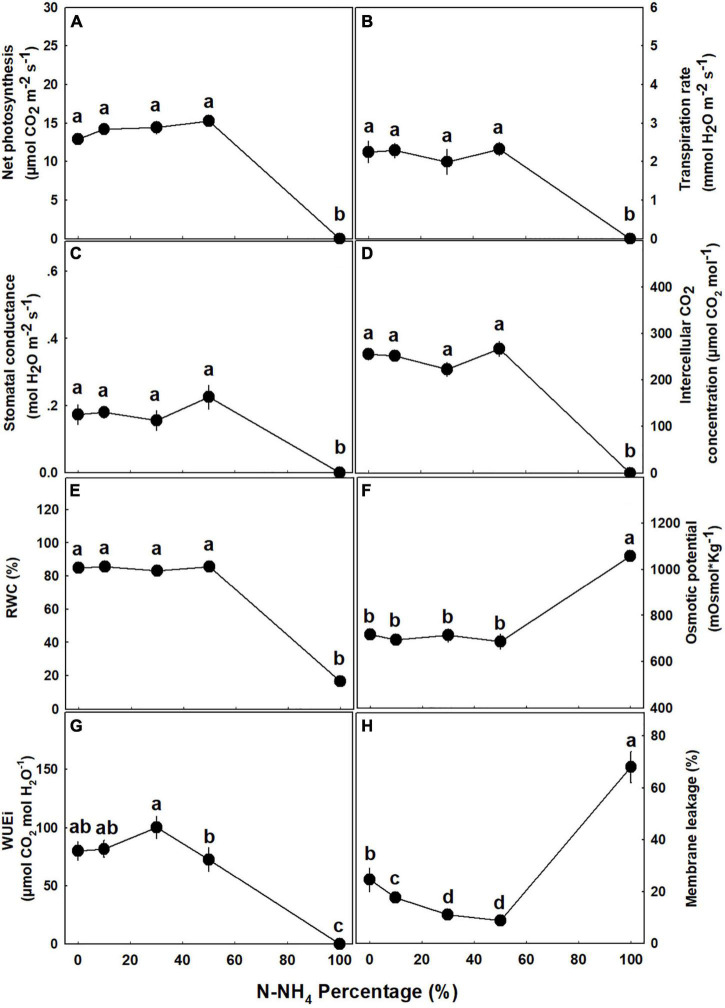
Effect of NH_4_/NO_3_ ratio on gas exchange, membrane leakage, and water relations parameters in medical cannabis plants. Net photosynthesis rate **(A)**, transpiration rate **(B)**, stomatal conductance **(C)**, intercellular CO_2_ concentration **(D)**, relative water content (RWC) **(E)**, osmotic potential **(F)**, intrinsic water use efficiency (WUEi) **(G)**, and membrane leakage **(H)**. Presented data are averages ± SE (*n* = 5). Different small letters above the means represent significant differences between treatments by Tukey HSD test at α = 0.05.

**FIGURE 9:**
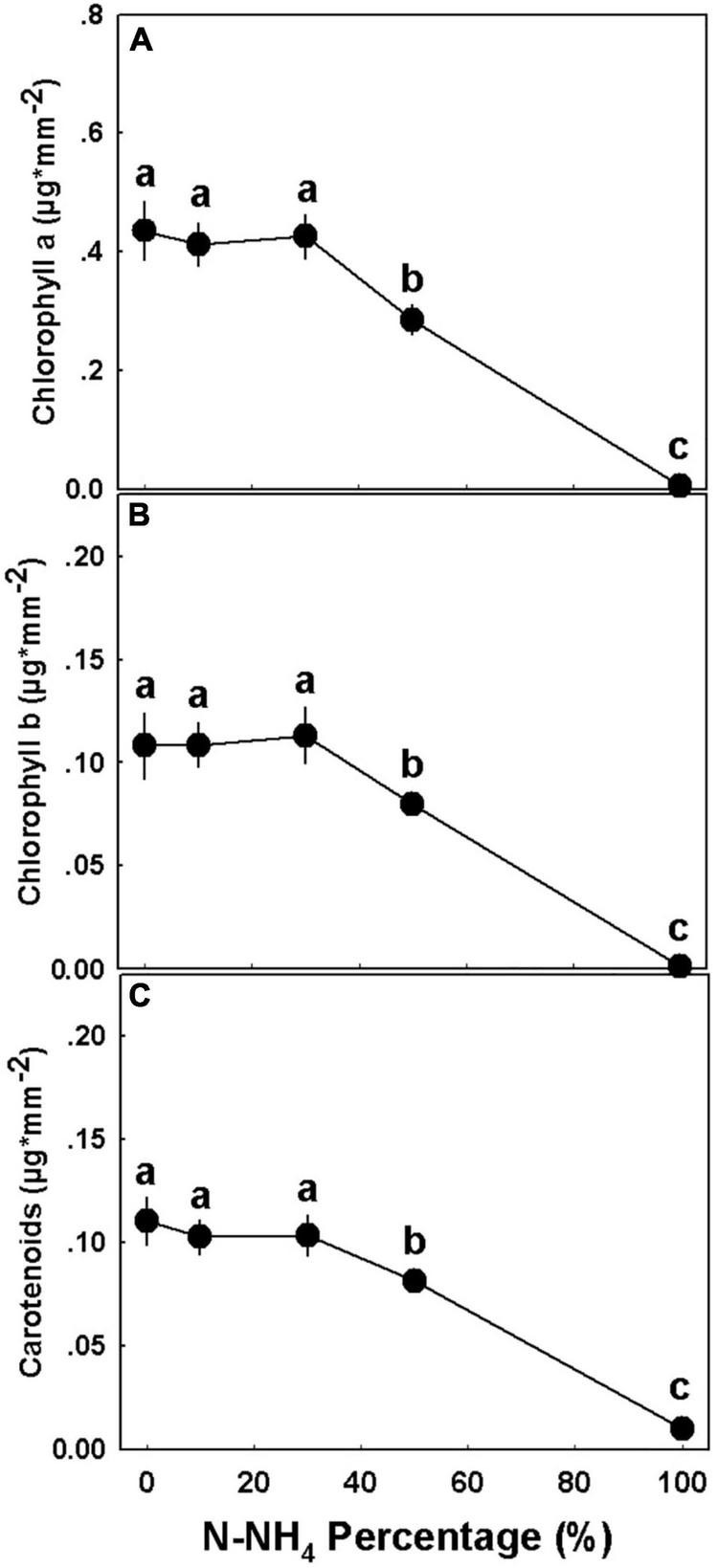
Effect of NH_4_/NO_3_ ratio on the concentration of photosynthetic pigments in medical cannabis plants. Chlorophyll a **(A)**, chlorophyll b **(B)**, and carotenoids **(C)**. Presented data are averages ± SE (*n* = 5). Different small letters above the means represent significant differences between treatments by Tukey HSD test at α = 0.05.

The percentage of NH_4_ form applied to the medical cannabis plants generated a bi-phasic physiological dose-response (Figure 8). At the concentration range of 0–50% NH_4_, most of the physiological parameters examined, including photosynthesis rate, transpiration rate, stomatal conductance, relative water content, osmotic potential, and water use efficiency, were optimal and indicated a proper plant function and a vital physiological state (Figure 8). At the second phase, under the high NH_4_ level of 100% NH_4_, a severe tissue damage was induced: Plant gas exchange ceased (Figures 8A–D,G), relative water content was very low (Figure 8E), osmotic potential and membrane leakage were very high (Figures 8F,H), and photosynthetic pigment content was close to zero (Figure 9). Excluding the 100% NH_4_ treatment, membrane leakage of the leaf tissue demonstrated a unique trend, as the leakage decreased with the elevation of NH_4_ percentage, marking a stabilization of low leakage in the range of 30–50% NH_4_ (Figure 8H). Contents of photosynthetic pigments in 50% NH_4_ plants were significantly lower than of plants receiving lower inputs of NH_4_, and significantly higher than of 100% NH_4_ plants (Figure 9).

## Discussion

Nitrogen nutrition is one of the major abiotic parameters affecting developmental, physiological, and metabolic processes in plants, including medical cannabis (Saloner and Bernstein, 2020, 2021). As the form of N taken up by the roots affects efficiency and metabolism of N in plants, we aimed to examine the response of medical cannabis to different NH_4_/NO_3_ ratios. The results reveal a significant sensitivity of medical cannabis plants to NH_4_/NO_3_ ratios. Plant development, physiological state, inflorescence yield, and secondary metabolite production were all best under NO_3_ nutrition when no NH_4_ was supplied. Under 10–30% NH_4_ supply, the plants did not demonstrate any external or internal toxicity symptoms but produced less yield. At 50% NH_4_, plants started to show toxicity injuries, whereas, at 100% NH_4_, the plants were substantially damaged by NH_4_ toxicity, which was observed already at early stages of the experiment. The results emphasize the significance of nutrition studies in medical cannabis for understanding the plant nutritional requirements and developing an optimal nutritional protocol.

Our results revealed that similar to many other plant species (Goyal and Huffaker, 1984; Wang and Li, 2004; Britto and Kronzucker, 2013; Boschiero et al., 2019), medical cannabis as well functions better under NO_3_ nutrition than NH_4_ nutrition. The plants which received the low NH_4_ levels (0–30% NH_4_) demonstrated the best physiological state (Figure 8), with high photosynthetic pigment contents (Figure 9), and consequently were larger and produced higher yield than plants that received higher concentrations of NH_4_ (Figure 3). Interestingly, although our latest studies concerning N nutrition in cannabis revealed a correlation between the plant N content and chlorophyll formation (Saloner and Bernstein, 2020, 2021), the data obtained in this study show a negative correlation between the two parameters, implying that high pigment production is better correlated with the plant physiological state than the tissue N content. High NO_3_ levels are known to increase rhizosphere pH (Crawford and Glass, 1998; Neumann and Römheld, 2012). In accordance, we obtained a significant increase in leachate pH in response to solely NO_3_ nutrition, while ratios of both NO_3_ and NH_4_ did not alter the pH (Supplementary Figure 2), marking a disadvantage for using NO_3_ as the sole N source, especially while growing hydroponically or in an inert media such as perlite.

NH_4_ toxicity is a well-documented phenomenon in plants (Goyal and Huffaker, 1984; Britto and Kronzucker, 2002; Roosta and Schjoerring, 2007). In the present study, plants that received only NH_4_ suffered from severe NH_4_ toxicity, as all developmental and physiological parameters measured were negatively affected, indicating an impaired plant function. The impact was so severe that gas exchange functions of the leaves ceased (Figures 8A–D,G), water content and photosynthetic pigment contents decreased substantially (Figures 3D, 8E, 9), and consequentially the osmotic potential was high and membrane stability was impaired (Figure 8F,H). This response induced a significant decrease in biomass production and development of visual toxicity symptoms (Figures 1–3), leading to plant death. These NH_4_ toxicity effects are in accord with previously reported NH_4_ toxicity effects in other plant species (reviewed by Britto and Kronzucker, 2002). At the last 2 weeks of the experiment, about 45 days after the initiation of the treatments, plants of the 50% NH_4_ treatment as well began to demonstrate toxicity symptoms, resulting in wilting of 40% of the plants in this treatment at the last week of the experiment. As the physiological measurements were conducted before the appearance of the toxicity damage at the last 2 weeks of the experiment, they are not reflected in the physiological data presented in Figure 8, and only the photosynthetic pigment content and the leaves biomass may indicate the initiation of the damage response (Figures 3B, 9). As was suggested by Britto and Kronzucker (2002), the toxicity damage caused by NH_4_ might result from over-time accumulation of NH_4_ or other metabolites in the plant tissue. This may explain why the commencement of plant damage occurred earlier under 100% NH_4_ than under 50% NH_4_ (Figure 2), showing that in *C. sativa* the NH_4_ toxicity damage is cumulative over time. An increase in % NH_4_ in the treatments entails an accompanied decrease in NO_3_, and vice versa. Therefore, the plant damage obtained under high NH_4_ (50–100% NH_4_) may reflect also effects of low or lack of NO_3_ supply. Similarly, the optimal plant function observed under 0% NH_4_ (and 100% NO_3_) may result also from the high NO_3_ supply, which increased uptake of essential minerals such as Mg and Ca (Figure 7), and enabled proper gas exchange and chlorophyll production (Figures 8, 9).

The mechanism for NH_4_ toxicity was widely investigated and elaborated on by many studies, and numerous possible negative influences of NH_4_ nutrition were considered. These include a decrease in chlorophyll content and hence photosynthetic activity, a reduction in carbohydrate production and the associated decrease in energy availability, increased proton efflux and unstable root pH homeostasis, oxidative stress, futile transmembrane NH_3_/NH_4_ cycling, increased accumulation of toxic NH_3_ and associated tissue damage, and decreased uptake and accumulation of vital cations (Goyal and Huffaker, 1984; Britto and Kronzucker, 2002, 2005; Roosta and Schjoerring, 2007; Neumann and Römheld, 2012; Coskun et al., 2013; Bittsánszky et al., 2015). The results we present in this study demonstrate that the NH_4_ toxicity response of cannabis is an integration of the mechanisms mentioned above: (i) Reduced contents of photosynthetic pigments (Figure 9) severely inhibited gas exchange parameters and photosynthesis (Figure 8). (ii) The substantial increase in membrane leakage (Figure 8H) indicates an oxidative damage induced by excess of NH_4_ or by a direct toxic effect of overexposure to NH_3_. (iii) A decrease in rhizosphere pH (Supplementary Figure 2) may damage root homeostasis, and decrease water uptake and leaf water content. (iv) The substantial decrease in tissue concentration of the essential macronutrients Ca and Mg (Figures 7E,F) likely caused by competition for uptake between NH_4_ and these cations, may exert deficiency damages, weakening the plants further.

The effects of NH_4_/NO_3_ ratio on the cannabis plant ionome that we report in this study are in accord with known cation-anion interactions for plant uptake and *in-planta* translocation. The results reveal a significant interaction between the N form supplied, and the ability of the cannabis plant to absorb and transport nutrients (Figure 7). First, NO_3_ accumulation in the plants increased with the increase in the concentration of NO_3_ supplied, demonstrating a relation between supply and accumulation of NO_3_. This confirms once again that different NH_4_/NO_3_ ratios induce changes in N accumulation. This was also apparent from the total N analysis, as total N concentration generally increased with the increase in NH_4_ supply, implying that higher NH_4_ promoted N uptake and translocation of N to the leaves and inflorescence, and thus has a direct effect on N metabolism. These results for N-NO_3_ and total N accumulation are supported by studies of other plant species (Haynes, 1986; Britto and Kronzucker, 2002, 2005; Ohyama, 2010; Zhang et al., 2019). The results show a considerable effect of NH_4_/NO_3_ ratio on Ca and Mg nutrition. As Ca and Mg are cations which may compete with NH_4_, their uptake, translocation and accumulation in the cannabis plants were negatively influenced by the increase in NH_4_ supply, and their concentration in the plant organs decreased significantly (Figures 7E,F), as was shown for other plant species (Errebhi and Wilcox, 1990a,b; Bernstein et al., 2005; Roosta and Schjoerring, 2007; Boschiero et al., 2019). Unexpectedly, the increased NH_4_ supply resulted in a moderate increase in K concentration in most plant organs (Figure 7C). Nevertheless, the overall amount of K in the entire plant decreased with the increase in NH_4_ supply (data not shown), and hence the total plant-level accumulation of K correlates with the well known competition for root uptake usually found between the two cations (Helali et al., 2010; Hawkesford et al., 2012; White, 2012). The concentration of P, which is mainly taken up in the form of the anion H_2_PO_4_^–^ (at the experimental pH range), generally decreased with the increase in NO_3_^–^ supply, marking an antagonistic anion-anion relationships between NO_3_^–^ and H_2_PO_4_^–^ uptake in cannabis, which was demonstrated for other plant species as well (Kinjo and Pratt, 1971; Feigin, 1990; Serna et al., 1992; Gniazdowska and Rychter, 2000). The concentration of the micronutrients Mn, Zn, Fe, and Cu was only moderately affected by the NH_4_/NO_3_ ratio, and the most pronounced responses, as well as the highest accumulation levels, were found in the roots (Figures 7G–J). The micronutrient accumulation results, showing highest accumulation in the roots and sensitivity to extreme shortage/toxicity of other minerals, matches the trends we reported previously for micronutrients accumulation in medical cannabis (Saloner et al., 2019; Saloner and Bernstein, 2020, 2021; Shiponi and Bernstein, 2021a).

### Effects of NH_4_/NO_3_ Ratio on Secondary Metabolism in Cannabis

The identified general response trend of many secondary metabolites to NH_4_ input in cannabis was highest production under low NH_4_ supply, and an overall reduction under elevation of NH_4_ supply (Figures 4–6 and Supplementary Figure 4). We consider two possible driving forces for this response trend: (i) Involvement of a stress response mechanism, and (ii) an effect of the availability of N in the plant tissues (following the “Carbon-nutrient balance hypothesis”).

i Considering involvement of a stress response: It was reported for numerous plant species, that an increase in plant secondary metabolism is part of a stress response, that is elicited by biotic or abiotic stresses (Gershenzon, 1984; Nascimento and Fett-Neto, 2010; Ramakrishna and Ravishankar, 2011; Gorelick and Bernstein, 2014; Jonathan et al., 2015; Sampaio et al., 2016; Sathiyabama et al., 2016). In accord with this notion, our previous studies with cannabis demonstrated stimulation of terpenoid and cannabinoid production under nutrient deficiency stress of N (Saloner and Bernstein, 2021) as well as P (Shiponi and Bernstein, 2021b). However, in the present case of medical cannabis response to NH_4_/NO_3_ ratio, the results revealed a negative rather than a positive correlation between secondary metabolism and plant stress. I.e., the concentration of secondary metabolites mostly decreased with the increase in NH_4_ supply, therefore not correlating with the plant’s overall stress response, which increased under high NH_4_ supply. As the physiological state of the plant was optimal under low inputs of NH_4_ (and high inputs of NO_3_), and these conditions overall promoted secondary metabolism, we conclude that cannabinoid and terpenoid production is not linked to the NH_4_-induced stress response.
ii Considering effect of N availability in the plant tissue: We have recently demonstrated that an optimal N nutrition, and high concentration of N in the plant, do not correlate with high secondary metabolism in cannabis (Saloner and Bernstein, 2021). Rather, we claimed, that the main factor governing the decrease in cannabinoid and terpenoid production is the tissue N content, which increase gradually with the increase in N supply (from 30 to 320 mg L^–1^) (Saloner and Bernstein, 2021). This suggestion, supported by results for numerous other plants (Bryant et al., 1983; Coley et al., 1985; Fritz et al., 2006; Palumbo et al., 2007; Massad et al., 2012; Albornoz, 2016), supports the carbon-nutrient balance hypothesis which states that under low N content production of N-rich primary metabolites and hence growth are restricted, and plant metabolism and energy expenditure shifts from creating N-containing metabolites to the production of metabolites that do not contain N (Iason and Hester, 1993; Lerdau and Coley, 2002; Rembiałkowska, 2007), such as cannabinoids and terpenoids. The results of the current (Figures 4–6) as well as the previous study into N nutrition of cannabis (Saloner and Bernstein, 2021), indeed confirm that the production of secondary metabolites in the cannabis inflorescence is highest under low N concentration in the inflorescence and in the plant, and decreases with the increase in inflorescence (and plant) N concentration (Figure 7A). As there is no known translocation and movement of cannabinoids and terpenoids in the plant, and these compounds are accumulated where they are formed, we suggest a specific impact of N in the inflorescence, creating a negative correlation between inflorescence N concentration and the production of secondary metabolites that do not contain N, such as cannabinoids and terpenoids.

Furthermore, the analysis of N-NO_3_ concentration in the plant tissue, enabled us to refine the proposed model, and suggest that total N concentration in the inflorescence, and not N-NO_3_ concentration, is the governing factor affecting cannabinoid and terpenoid biosynthesis. This is deduced from the lack of a consistent correlation trend between N-NO_3_ concentration and cannabis secondary metabolism in the cannabis inflorescence (Figure 7B; Saloner and Bernstein, 2021).

## Conclusion

The response of medical cannabis to different NH_4_/NO_3_ ratios was analyzed. The highest inflorescence yield and secondary metabolite contents were obtained under sole NO_3_ supply. It is therefore proposed that N-NO_3_ nutrition is optimal for medical cannabis plant function. Nevertheless, as moderate levels of 10–30% NH_4_ (20–60 ppm NH_4_) did not induce physiological damage, had only little adverse effect on the inflorescence and cannabinoid yield, and prevented pH changes and leaf tip burns caused by only NO_3_ nutrition, it seems safe to suggest to utilize these ratios for medical cannabis cultivation. As NH_4_ toxicity damage appeared under 50% NH_4_ nutrition (100 ppm NH_4_), and severe toxicity symptoms and plant death were obtained under the 100% NH_4_ regime, it is not recommended to use levels higher than 30% NH_4_ for medical cannabis cultivation. The results suggest that total N concentration in the inflorescence, and not N-NO_3_ concentration, is the governing factor affecting cannabinoid and terpenoid biosynthesis.

Similar to other crop species, indications for some genotypic variability in plant response to mineral supply in available for cannabis (Yep et al., 2020; Shiponi and Bernstein, 2021b). This suggest that some fine-tuning may be required by the growers for adjusting N supply and NH_4_/NO_3_ ratios for specific cultivars.

## Data Availability Statement

The original contributions presented in the study are included in the article/Supplementary Material.

## Author Contributions

NB planned the experiments. AS carried out the experiments. Both authors wrote the manuscript, and approved the submitted version.

## Conflict of Interest

The authors declare that the research was conducted in the absence of any commercial or financial relationships that could be construed as a potential conflict of interest.

## Publisher’s Note

All claims expressed in this article are solely those of the authors and do not necessarily represent those of their affiliated organizations, or those of the publisher, the editors and the reviewers. Any product that may be evaluated in this article, or claim that may be made by its manufacturer, is not guaranteed or endorsed by the publisher.
