# Supplementary material for: Nitrogen Source Matters: High NH4/NO3 Ratio Reduces Cannabinoids, Terpenoids, and Yield in Medical Cannabis
Source: Front Plant Sci. 2022 Jun 1;13:830224. doi: 10.3389/fpls.2022.830224 (PMC9198551; doi:10.3389/fpls.2022.830224)
Supplement: Supplementary file 1 [file Data_Sheet_1.pdf]

Supplemental Material

Nitrogen source matters: High NH_4/NO_3 ratio reduces cannabinoids, terpenoids, and yield in medical cannabis

Avia Saloner, Nirit Bernstein*

Institute of Soil Water and Environmental Sciences, Volcani Center, 68 HaMaccabim Road P.O.B 15159 Rishon LeZion, 7505101, Israel.

Corresponding author: Nirit Bernstein Nirit@agri.gov.il

Supplumental Table 1: Concentrations of NH_4, and NO_3 in the experimental treatments*.

Treatment (% NH_4)**	NH_4 Concentration (mg L^{-1})	NO_3 Concentration (mg L^{-1})
**0**	0	200
**10**	20	180
**30**	60	140
**50**	100	100
**100**	200	0

* Concentrations of the other nutrients in the irrigation solution (in mM): 2.8 K^+, 1.9 P-PO_4^{2-}, 1.42 Ca^{2+}, 1.55 Mg^{2+}, 3.5 S-SO_4^{2-}, 1.7 Na^+, 1.43 Cl^-, 0.024 Fe^{2+}, 0.011 Mn^{2+}, 0.009 B^{3+}, 0.005 Zn^{2+}, 0.002 Cu^{2+}, and 0.0003 Mo^{2+}.

**Electrical conductivity (EC) of the irrigation solution was 1.7, 1.7, 1.8, 2.1, 2.5 (dS m^{-1}) for the 0, 10, 30, 50, 100 %NH4 treatments, respectively.

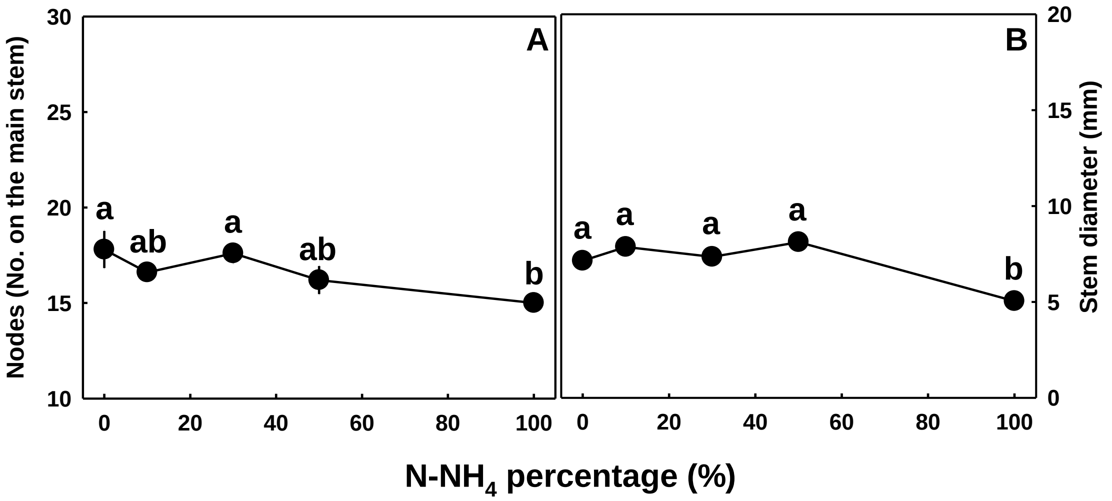

Supplementary Figure 1. Effect of NH_4/NO_3 ratio on the development of medical cannabis plants at the reproductive growth phase. Number of nodes on the main stem (**A**) and stem diameter (**B**). Presented data are averages ± SE (n=5). Different small letters above the means represent significant differences between treatments by Tukey HSD test at α=0.05.

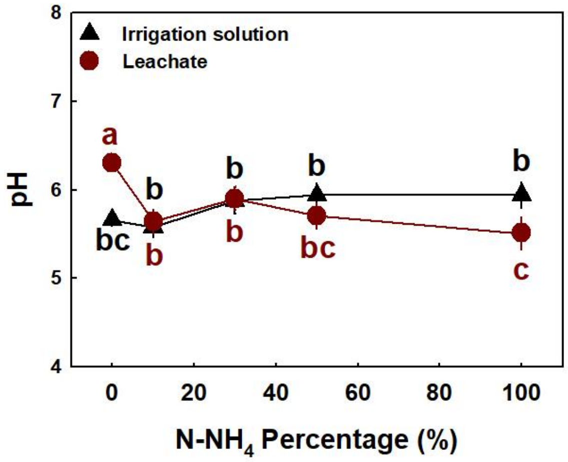

Supplementary Figure 2. Mean pH values of the irrigation and leachate solutions. Different small letters above the means represent significant differences by Tukey HSD test at α=0.05.

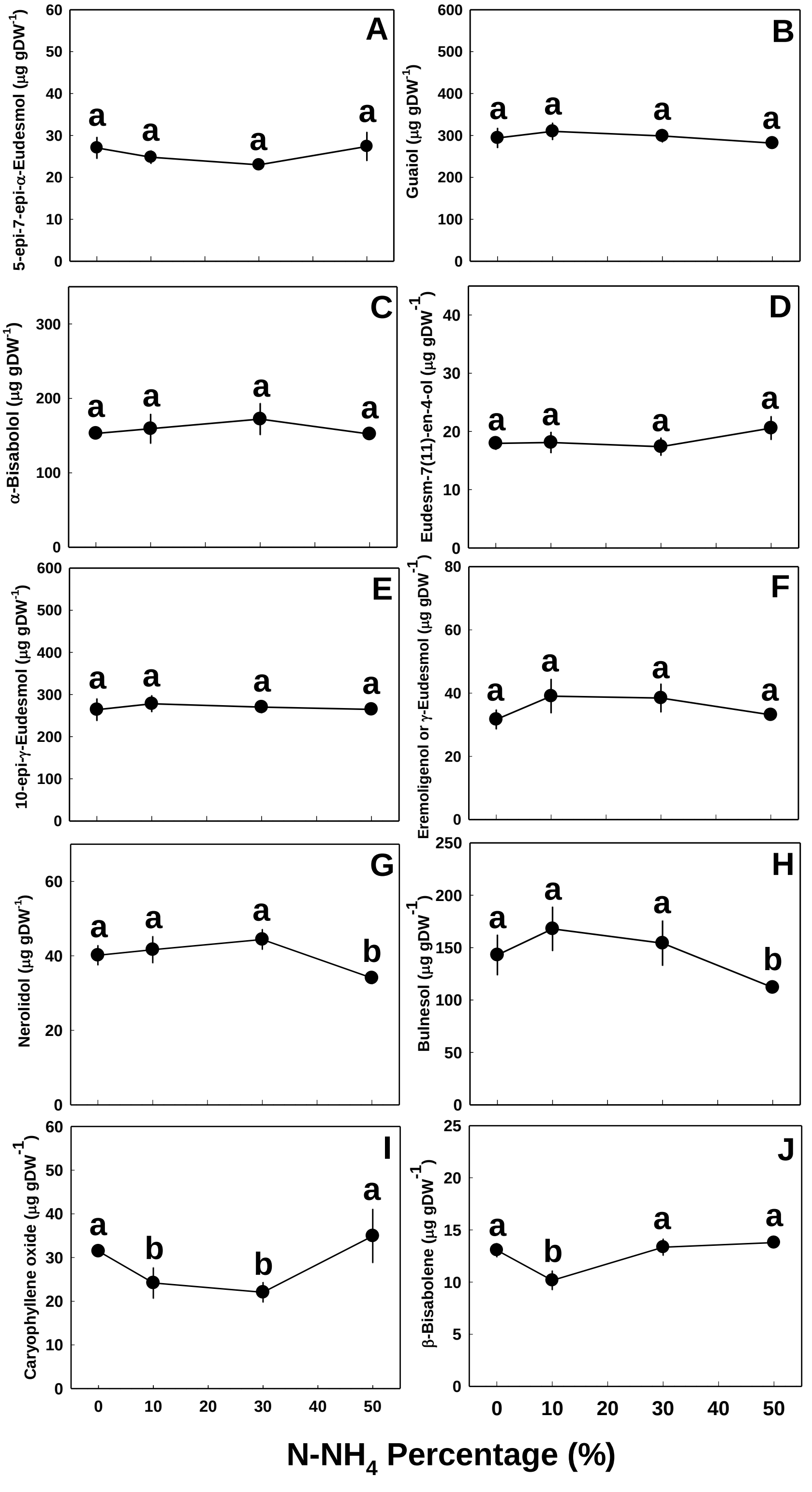

<u>Supplementary Figure 3</u>. Effect of NH_4/NO_3 ratio on sesquiterpene concentration in the top inflorescence of medical cannabis plants. 5-epi-7-epi-α-Eudesmol (**A**), guaiol (**B**), α-bisabolol (**C**), eudesm-7(11)-en-4-ol (**D**), 10-epi-γ-eudesmol (**E**), eremoligenol or γ-eudesmol (**F**), nerolidol (**G**), bulnesol (**H**), caryophyllene oxide (**I**), and β-bisabolene (**J**). Presented data are averages ± SE (n=5). Different small letters above the means represent significant differences between treatments by Tukey HSD test at α=0.05.

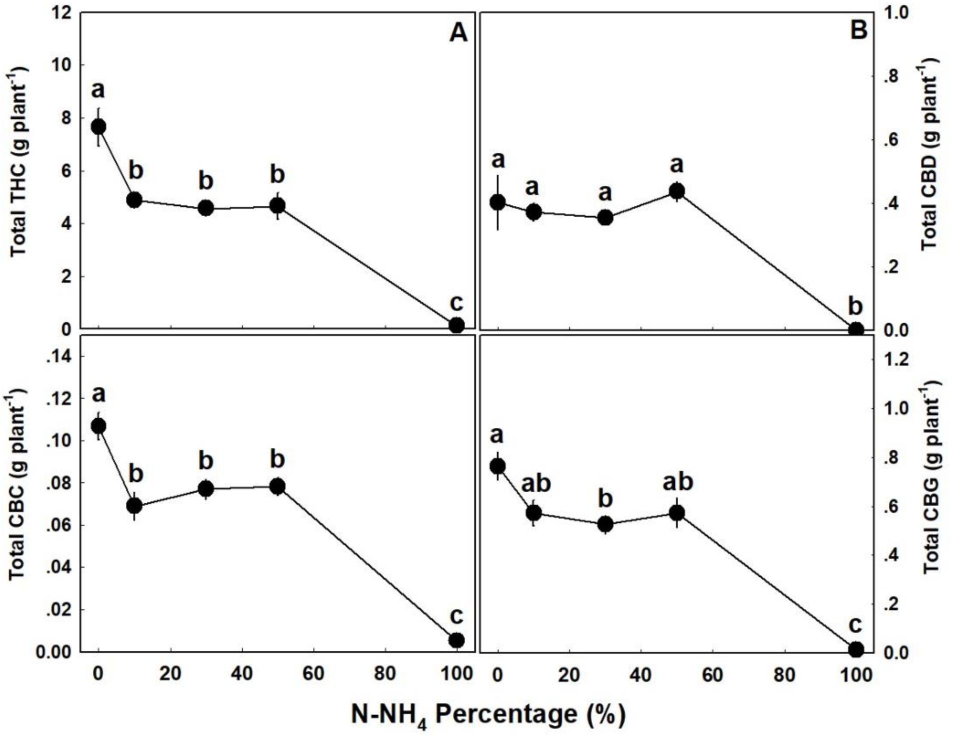

<u>Supplementary Figure 4</u>. Effect of NH_4/NO_3 on production of cannabinoids in medical cannabis plants. The total THC (**A**), CBD (**B**), CBC (**C**), and CBG (**D**) produced per plant. Total weight of each cannabinoid was calculated from the concentrations of the carboxylated plus the decarboxylated forms as is detailed in the material and methods section. Presented data are averages ± SE (n=5). Different small letters above the means represent significant differences between treatments by Tukey HSD test at α=0.05.
